# Association between neck and shoulder pain, back pain, low back pain and body composition parameters among the Japanese general population

**DOI:** 10.1186/s12891-015-0759-z

**Published:** 2015-11-04

**Authors:** Yoichi Iizuka, Haku Iizuka, Tokue Mieda, Tsuyoshi Tajika, Atsushi Yamamoto, Takashi Ohsawa, Tsuyoshi Sasaki, Kenji Takagishi

**Affiliations:** Department of Orthopaedic Surgery, Gunma University Graduate School of Medicine, 3-39-22, Showa, Maebashi, Gunma 371-8511 Japan

**Keywords:** Neck and shoulder pain, Back pain, Low back pain, Body composition, General population

## Abstract

**Background:**

Neck and shoulder pain, back pain and low back pain are common symptoms in Japanese subjects, and it is important to elucidate the pathology and associated factors of these pains due to their frequency and impact on the quality of life (QOL) and activities of daily living (ADL). The purpose of the present study was to investigate whether body composition is associated with these pains.

**Methods:**

We collected the data of 273 Japanese subjects regarding the presence and the visual analogue scale (VAS) of neck and shoulder pain, back pain, low back pain and body composition parameters calculated using bioelectrical impedance analysis (BIA) technology. Furthermore, we investigated the association between these pains and the body composition using statistical methods.

**Results:**

According to a multivariate analysis adjusted for age and gender, lower total body water ratio was significantly associated with the presence of neck and shoulder pain at present (*P* < 0.05); additionally, total body muscle mass (standardized β = −0.26, 95 % CI, −0.17 - -0.008, *P* < 0.05), total body water (standardized β = −0.27, 95 % CI, −0.23 - -0.04, *P* < 0.01), appendicular muscle mass (standardized β = −0.29, 95 % CI, −0.36 - -0.04, *P* < 0.05), and the appendicular muscle mass index (AMI) (standardized β = −0.24, 95 % CI, −1.18 - -0.20, *P* <0.01) were negatively correlated with the VAS of neck and shoulder pain, whereas no body composition parameters were significantly associated with back pain, low back pain at present and any type of chronic pain.

**Conclusions:**

The present study demonstrated that some body composition parameters regarding body water and body muscle were associated or correlated with the presence or intensity of neck and shoulder pain.

## Background

Neck and shoulder pain, back pain and low back pain are common symptoms in the clinical setting. In a comprehensive survey of the living conditions of the Japanese population, the most common subjective symptom in Japanese males was low back pain followed by neck and shoulder pain (*katakori* in Japanese [1–7]), and the most common symptom in Japanese females was neck and shoulder pain followed by low back pain [8]. Takasawa et al. reported that the prevalence of neck and shoulder pain in the Japanese general population was 48.3 % according to the data of medical checkups for the general population [5]. Yoshimura et al. surveyed the prevalence of motor system organs and reported that the prevalence of low back pain in the Japanese general population was 37.7 % [9]. Furthermore, musculoskeletal pain (including neck and shoulder pain, back pain and low back pain) has been reported to be associated with a decreased quality of life (QOL) and activities of daily living (ADL) [10–13]. Therefore, it is crucial to elucidate the pathology and associated factors of such pain in terms of their frequency and impact on daily living, and numerous studies has been reported to elucidate the pathology and associated factors of these types of pain to date. However, much about the pathology and associated factors of such pain remains unclear.

The association between body composition including muscle and fat mass and several health problems has recently been reported and several lines of evidence have been obtained regarding the role of body composition in several health problems. For instance, age-related muscle loss with an increased fat mass has been reported to be associated with increased risks of disability and mortality [14, 15]. Furthermore, the maintenance of muscle mass has also been reported to be associated with a decreased risk of cardiovascular diseases, including dyslipidemia and diabetes [16–18].

On the other hand, some researchers reported the association of body composition with musculoskeletal pain, and they demonstrated that a greater fat mass and an attenuated muscle mass as body composition factors are associated with musculoskeletal pain [19–26]. However, to the best of our knowledge, no studies have so far investigated the association of body composition with such musculoskeletal pain in the Japanese general population, including neck and shoulder pain, termed *katakori* in Japanese, in which the precise pathology has not been determined and treatment strategies have not been established, despite its prevalence.

The purpose of the present cross-sectional study is to determine the association between neck and shoulder pain, back pain, low back pain and body composition in the Japanese general population.

## Methods

### Ethical statement

The present study was approved by the Institutional Review Board of Gunma University. Written informed consent was obtained from all subjects.

### Study subjects

In 2014, annual medical examinations concerning life-related diseases were held in Katashina village (population: 4465 in 2014) in Gunma prefecture in Japan, and the total number of subjects examined was 1069. Of those 1069 examinees, 432 participated in the musculoskeletal examinations, including a questionnaire survey concerning neck and shoulder pain, back pain and low back pain and an analysis of the body composition.

**Fig. 1:**
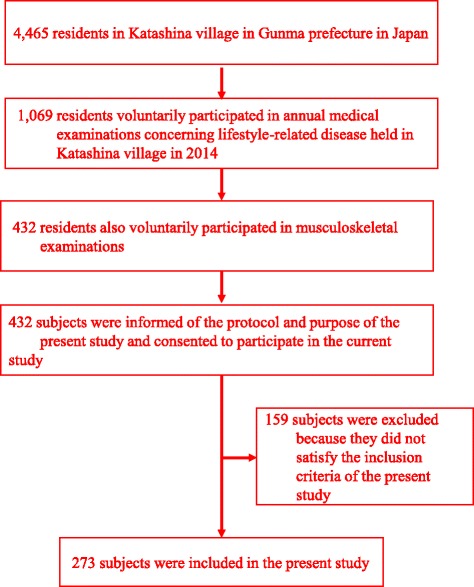
Selection of the Study Population

The inclusion criteria for the present study were as follows: (1) individuals whose demographic data, including age and gender, were recorded, (2) individuals who answered the questionnaire regarding musculoskeletal pain, including neck and shoulder pain, back pain and low back pain, (3) individuals who underwent body composition examinations using the Tanita MC-780 body composition analyzer (Tanita Corp., Tokyo, Japan), and (4) individuals who were informed of the protocol and purpose of the current study and consented to participate. According to these criteria, a total of 273 subjects (94 males and 179 females, mean age: 64.3 years, range: 23–90) were included in the current study (Fig. 1).

### Questionnaire survey regarding neck and shoulder pain, back pain and low back pain

The questionnaire used in the present study is shown in Table 1. The subjects completed questionnaires regarding the presence of present pain, such as neck and shoulder pain, back pain and low back pain, and they also completed questionnaires regarding the presence of chronic pain, such as chronic neck and shoulder pain, chronic back pain and chronic low back pain persisting for 3 months or more. Furthermore, the subjects completed questionnaires regarding the intensity of each type of present pain based on a visual analogue scale (VAS, measured along a distance of 0–100 mm). Six orthopedic surgeons asked the subjects to complete the questionnaires. Although the questionnaires were basically in a self-administered mode, an interviewer-administered mode was also used by these six orthopedic surgeons if the subjects could not understand the meaning of the questions. The interviewers, that is, the six orthopedic surgeons, were trained by the first author (IY) to ensure standardization.

**Table 1:**
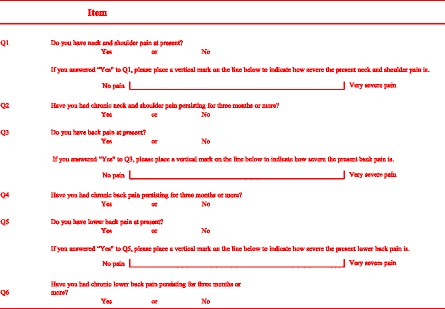
The items of the questionnaire regarding the presence of present pain, chronic pain and pain intensity at present

### Body composition measurements

Regarding the body composition analysis, the Tanita MC-780 body composition analyzer, which analyzes the body composition using bioelectrical impedance analysis (BIA) technology [27–31], was used. BIA can assess body composition parameters by assessing the difference of impedance of each component of the body, including muscle and lean tissue. The Tanita MC-780 body composition analyzer used in the present study assesses the body composition using a constant current source with a high-frequency current (50 kHz, 90 μA). After the subjects stood on the platform with the electrodes of the analyzer and grasped the handgrip of the electrodes with both hands, several body composition parameters including total body muscle mass (kg), total body fat mass (kg), total body water (kg), total body fat ratio (%), total body water ratio (%), muscle mass of the arms and the legs (kg), body mass index (BMI) and basal metabolic rate (BMR) were automatically calculated. In several previous studies, BIA was shown to be a reliable and valid technology [27–31].

Appendicular muscle mass (kg) was calculated as the sum of the muscle mass of the arms and legs, and the appendicular muscle mass index (AMI) was also calculated using the following formula: AMI (kg/m^2^) = appendicular muscle mass (kg) / body height^2^ (m^2^) [32–34].

### Statistical analyses

Univariate and multivariate logistic regression models were used to explore the association between present pain or chronic pain and body composition parameters. In these models, the odds ratio (OR) and the 95 % confidence interval (CI) were calculated. Furthermore, simple and multiple linear regression models were also used to explore the association between the VAS of present pain and body composition parameters. In these models, standardized β and 95 % CI were calculated. In each univariate model for the presence of pain or the VAS of present pain as a dependent variable, only one parameter was selected as an explanatory variable to assess the crude association between body composition parameters and the presence of pain or the VAS of present pain. In each multivariate model for the presence of pain or the VAS of present pain as a dependent variable, not only one body composition parameter but also age and gender were selected as explanatory variables, since age and gender were assumed to be possible confounders. The statistical assumptions for the regression analysis were verified using a residual analysis. The results were not corrected for multiple testing, since the present study is an exploratory study and each of the individual associations was considered to be of interest. The level of significance was set at *p* < 0.05. All statistical analyses were performed using the IBM SPSS Statistics 22 software package (IBM Japan, Tokyo, Japan).

## Results

### Prevalence of neck and shoulder pain, back pain and low back pain

The characteristics of the 273 patients who were included in the present study are demonstrated in Table 2. Regarding present pain, the prevalence of neck and shoulder pain, back pain, and low back pain were observed in 55.6 %, 27.4 % and 61.5 % of all subjects, respectively. In addition, regarding chronic pain, the prevalence of neck and shoulder pain, back pain and low back pain were observed in 23.0 %, 9.1 % and 22.7 % of all subjects, respectively. Furthermore, the VAS scores of neck and shoulder pain, back pain and low back pain at present were 24.1 ± 28.2, 10.3 ± 20.7 and 22.9 ± 25.1, respectively.

**Table 2:** Summary of the surveyed variables of the 273 subjects

	Values
Age (y)	64.3 ± 13.2
Gender (F/M)	179/94
Total body muscle mass (kg)	39.6 ± 8.1
Total body fat mass (kg)	15.2 ± 5.9
Total body water (kg)	30.2 ± 5.5
Total body fat ratio (%)	26.5 ± 8.4
Total body water ratio (%)	53.1 ± 5.1
Appendicular muscle mass (kg)	17.1 ± 4.0
Appendicular fat mass (kg)	6.7 ± 2.3
Trunk muscle mass (kg)	22.4 ± 4.3
Trunk fat mass (kg)	8.5 ± 3.7
BMI (kg/m2)	23.4 ± 2.9
AMI (kg/m2)	6.9 ± 1.0
BMR (kcal)	1179.2 ± 216.5
Present pain	
neck and shoulder pain (n, %)	152, 55.6
VAS of neck and shoulder pain (mm)	24.1 ± 28.2
back pain (n, %)	75, 27.4
VAS of back pain (mm)	10.3 ± 20.7
Low back pain (n, %)	168, 61.5
VAS of low back pain (mm)	22.9 ± 25.1
Chronic pain	
Neck and shoulder pain (n, %)	63, 23.0
Back pain (n, %)	25, 9.1
Low back pain (n, %)	62, 22.7

BMI, body mass index; AMI, appendicular muscle index; BMR, basal metabolic rate; VAS, visual analogue scale

### The association between the presence of pain and body composition

The unadjusted and adjusted analyses with logistic regression models for present pain are shown in Table 3. In the unadjusted analysis with the univariate logistic regression model, lower age (OR = 0.96 , 95 % CI, 0.94 - 0.98, *P* < 0.001), female gender (OR = 1.84, 95 % CI, 1.11 - 3.05, *P* < 0.05), higher total body fat ratio (OR = 1.03, 95 % CI, 1.00 - 1.06, *P* < 0.05), lower total body water ratio (OR = 0.93, 95 % CI, 0.88 - 0.97, *P* < 0.01) and higher appendicular fat mass (OR = 1.12, 95 % CI, 1.00 - 1.24, *P* < 0.05) were significantly associated with the presence of neck and shoulder pain, and lower age (OR = 0.98, 95 % CI, 0.96 - 0.99, *P* < 0.05) was significantly associated with the presence of back pain, whereas no parameters were significantly associated with low back pain. However, in the age- and gender-adjusted analysis with the multivariate logistic regression model, only lower total body water ratio (OR = 0.94, 95 % CI, 0.88-0.99, *P* < 0.05) was significantly associated with the presence of neck and shoulder pain, whereas no significant association was observed between the presence of back pain or low back pain and body composition parameters.

**Table 3:** Association between present pain and body composition parameters

	Model 1^a^			Model 2^b^		
	OR	95 % CI	*P*-value*	OR	95 % CI	*P*-value*
Neck and shoulder pain						
Age (y)	0.96	0.94 - 0.98	**<0.001**	-	-	-
Gender (F/M)	1.84	1.11 - 3.05	**<0.05**	-	-	-
Total body muscle mass (kg)	0.98	0.95 - 1.01	0.24	0.98	0.92 - 1.05	0.74
Total body fat mass (kg)	1.03	0.99 - 1.07	0.11	1.02	0.97 - 1.06	0.32
Total body water (kg)	0.96	0.92 - 1.00	0.11	0.96	0.89 - 1.03	0.27
Total body fat ratio (%)	1.03	1.00 - 1.06	**<0.05**	1.02	0.98 - 1.06	0.16
Total body water ratio (%)	0.93	0.88 - 0.97	**<0.01**	0.94	0.88 - 0.99	**<0.05**
Appendicular muscle mass (kg)	0.50	0.92 - 1.04	0.50	0.97	0.86 - 1.10	0.71
Appendicular fat mass (kg)	1.12	1.00 - 1.24	**<0.05**	1.05	0.93 - 1.18	0.38
Trunk muscle mass (kg)	0.95	0.90 - 1.01	0.11	0.98	0.88 - 1.10	0.82
Trunk fat mass (kg)	1.04	0.97 - 1.11	0.24	1.59	0.93 - 2.72	0.08
BMI (kg/m2)	0.98	0.91 - 1.07	0.78	1.01	0.93 - 1.10	0.75
AMI (kg/m2)	0.83	0.65 - 1.06	0.14	0.80	0.55 - 1.17	0.26
BMR (kcal)	1.00	0.99 - 1.00	0.62	1.00	0.99 - 1.00	0.95
Back pain						
Age (y)	0.98	0.96 - 0.99	**<0.05**	-	-	-
Gender (F/M)	1.07	0.61 - 1.87	0.81	-	-	-
Total body muscle mass (kg)	1.00	0.97 - 1.03	0.82	0.99	0.92 - 1.06	0.86
Total body fat mass (kg)	0.35	0.96 - 1.05	0.85	1.00	0.95 - 1.05	0.84
Total body water (kg)	0.99	0.95 - 1.04	0.88	0.97	0.90 - 1.05	0.51
Total body fat ratio (%)	1.00	0.97 - 1.03	0.66	1.01	0.97 - 1.06	0.45
Total body water ratio (%)	0.97	0.99 - 1.02	0.29	0.96	0.90 - 1.02	0.24
Appendicular muscle mass (kg)	1.01	0.95 - 1.08	0.66	0.98	0.86 - 1.12	0.83
Appendicular fat mass (kg)	1.02	0.91 - 1.13	0.73	1.00	0.88 - 1.14	0.93
Trunk muscle mass (kg)	1.00	0.94 - 1.06	0.99	0.99	0.87 - 1.12	0.91
Trunk fat mass (kg)	1.00	0.93 - 1.07	0.93	0.98	0.54 - 1.77	0.96
BMI (kg/m2)	1.05	0.80 - 1.36	0.99	1.01	0.92 - 1.10	0.80
AMI (kg/m2)	0.99	0.85 - 1.16	0.98	1.01	0.67 - 1.51	0.96
BMR (kcal)	1.00	0.99 - 1.00	0.71	1.00	0.99 - 1.00	0.82
Low back pain						
Age (y)	1.00	0.98 - 1.02	0.57	-	-	-
Gender (F/M)	0.80	0.47 - 1.35	0.40	-	-	-
Total body muscle mass (kg)	1.01	0.98 - 1.04	0.53	1.00	0.94 - 1.06	0.63
Total body fat mass (kg)	0.98	0.94 - 1.02	0.32	1.02	0.94 - 1.02	0.43
Total body water (kg)	1.00	0.96 - 1.05	0.76	0.98	0.91 - 1.06	0.72
Total body fat ratio (%)	0.98	0.95 - 1.01	0.32	0.98	0.94 - 1.02	0.48
Total body water ratio (%)	1.01	0.96 - 1.06	0.59	1.00	0.95 - 1.06	0.84
Appendicular muscle mass (kg)	1.01	0.95 - 1.08	0.59	1.00	0.89 - 1.13	0.89
Appendicular fat mass (kg)	0.95	0.86 - 1.05	0.35	0.96	0.86 - 1.08	0.55
Trunk muscle mass (kg)	1.02	0.96 - 1.08	0.49	1.00	0.88 - 1.12	0.93
Trunk fat mass (kg)	0.96	0.90 - 1.03	0.31	0.97	0.90 - 1.03	0.37
BMI (kg/m2)	1.00	0.79 - 1.28	0.34	0.95	0.88 - 1.03	0.28
AMI (kg/m2)	1.01	0.87 - 1.16	0.89	0.89	0.62 - 1.29	0.56
BMR (kcal)	1.00	0.99 - 1.00	0.70	1.00	0.99 - 1.00	0.91

BMI, body mass index; AMI, appendicular muscle index; BMR, basal metabolic rate; OR, odds ratio; CI, confidence interval. ^a^Univariate logistic regression model; ^b^Multivariate logistic regression model adjusted for age and gender; *Significant *P*-values are in bold

Conversely, no significant association was observed between the presence of chronic pain and any body composition parameters in the analyses using the logistic regression model for chronic pain (Table 4).

**Table 4:** Association between chronic pain and body composition parameters

	Model 1^a^			Model 2^b^		
	OR	95 % CI	*P*-value*	OR	95 % CI	*P*-value*
Chronic neck and shoulder pain						
Age (y)	0.97	0.95 - 0.99	**<0.01**	-	-	-
Gender (F/M)	1.93	1.01 - 3.67	**<0.05**	-	-	-
Total body muscle mass (kg)	0.98	0.94 - 1.02	0.38	1.02	0.94 - 1.10	0.54
Total body fat mass (kg)	1.01	0.96 - 1.06	0.59	1.03	0.97 - 1.10	0.27
Total body water (kg)	0.97	0.92 - 1.02	0.35	1.01	0.92 - 1.10	0.81
Total body fat ratio (%)	1.01	0.97 - 1.04	0.56	0.98	0.94 - 1.03	0.63
Total body water ratio (%)	0.98	0.92 - 1.03	0.48	1.01	0.92 - 1.10	0.81
Appendicular muscle mass (kg)	0.97	0.90 - 1.04	0.49	1.01	0.87 - 1.16	0.87
Appendicular fat mass (kg)	1.07	0.95 - 1.20	0.22	1.00	0.88 - 1.15	0.89
Trunk muscle mass (kg)	0.96	0.90 - 1.03	0.31	1.06	0.93 - 1.22	0.36
Trunk fat mass (kg)	1.00	0.93 - 1.08	0.94	0.99	0.91 - 1.07	0.89
BMI (kg/m^2^)	0.96	0.87 - 1.06	0.48	0.98	0.89 - 1.08	0.81
AMI (kg/m^2^)	0.83	0.62 - 1.11	0.22	0.87	0.55 - 1.37	0.57
BMR (kcal)	1.00	0.99 - 1.00	0.66	1.00	0.99 - 1.00	0.58
Chronic back pain						
Age (y)	0.98	0.95 - 1.01	0.45	-	-	-
Gender (F/M)	1.39	0.55 - 3.45	0.47	-	-	-
Total body muscle mass (kg)	0.99	0.94 - 1.04	0.82	1.02	0.92 - 1.14	0.63
Total body fat mass (kg)	0.99	0.93 - 1.06	0.94	0.98	0.91 - 1.06	0.77
Total body water (kg)	0.98	0.91 - 1.06	0.78	1.01	0.89 - 1.14	0.83
Total body fat ratio (%)	1.00	0.95 - 1.05	0.86	0.99	0.93 - 1.05	0.81
Total body water ratio (%)	0.99	0.91 - 1.07	0.79	1.00	0.91 - 1.09	0.95
Appendicular muscle mass (kg)	0.99	0.89 - 1.09	0.85	1.02	0.84 - 1.26	0.78
Appendicular fat mass (kg)	1.02	0.86 - 1.20	0.81	0.98	0.81 - 1.19	0.88
Trunk muscle mass (kg)	0.98	0.89 - 1.08	0.81	1.05	0.87 - 1.28	0.56
Trunk fat mass (kg)	0.98	0.88 - 1.10	0.79	0.97	0.87 - 1.10	0.71
BMI (kg/m^2^)	0.96	0.84-1.11	0.63	0.97	0.84 - 1.12	0.73
AMI (kg/m^2^)	0.9	0.59 - 1.38	0.63	0.93	0.49 - 1.77	0.84
BMR (kcal)	1.00	0.99 - 1.00	0.91	1.00	0.99 - 1.00	0.72
Chronic low back pain						
Age (y)	1.00	0.98 - 1.02	0.75	-	-	-
Gender (F/M)	0.65	0.36 - 1.17	0.15	-	-	-
Total muscle mass (kg)	1.02	0.98 - 1.05	0.19	1.01	0.94 - 1.08	0.74
Total fat mass (kg)	0.97	0.92 - 1.02	0.30	0.98	0.93 - 1.03	0.54
Total body water (kg)	1.03	0.98 - 1.08	0.19	1.01	0.93 - 1.10	0.66
Total fat ratio (%)	0.97	0.94 - 1.00	0.11	0.97	0.93 - 1.02	0.34
Total body water ratio (%)	1.04	0.98 - 1.10	0.11	1.03	0.97 - 1.09	0.29
Appendicular muscle mass (kg)	1.04	0.97 - 1.12	0.18	1.03	0.90 - 1.18	0.59
Appendicular fat mass (kg)	0.94	0.83 - 1.06	0.34	0.97	0.85 - 1.12	0.72
Trunk muscle mass (kg)	1.04	0.97 - 1.10	0.22	1.00	0.88 - 1.14	0.95
Trunk fat mass (kg)	0.95	0.88 - 1.03	0.29	0.96	0.89 - 1.05	0.44
BMI (kg/m^2^)	0.97	0.88 - 1.07	0.56	0.96	0.87 - 1.06	0.96
AMI (kg/m^2^)	0.28	0.88 - 1.53	0.36	1.03	0.68 - 1.57	0.85
BMR (kcal)	1.00	0.99- 1.00	0.25	1.00	0.99 - 1.00	0.78

BMI, body mass index; AMI, appendicular muscle index; BMR, basal metabolic rate; OR, odds ratio; CI, confidence interval. ^a^Univariate logistic regression model; ^b^Multivariate logistic regression model adjusted for age and gender; *Significant *P*-values are in bold

### The association between the intensity of present pain and body composition

The unadjusted and adjusted analyses with linear regression models for the VAS of pain at present are shown in Table 5. In the unadjusted analysis with the simple linear regression model, age (standardized β = −0.16, 95 % CI, −0.06 - -0.01, *P* < 0.01), total body muscle mass (standardized β = −0.13, 95 % CI, −0.09 - -0.007, *P* < 0.05), total body water (standardized β = −0.17, 95 % CI, −0.15 - -0.03, *P* < 0.01), the total body water ratio (standardized β = −0.15, 95 % CI, −0.14 - -0.01, *P* < 0.05), appendicular muscle mass (standardized β = −0.13, 95 % CI, −0.17 - -0.008, *P* < 0.001), trunk muscle mass (standardized β = −0.13, 95 % CI, −0.16 - -0.01, *P* < 0.05) and the AMI (standardized β = −0.16, 95 % CI, −0.81 - -0.14, *P* < 0.01) were negatively correlated with the VAS of neck and shoulder pain, whereas no significant correlation was observed between back pain or low back pain and any body composition parameters, although the BMR was found to be close to the limit for significance. Furthermore, in the adjusted analysis for age and gender with the multiple linear regression model, total body muscle mass (standardized β = −0.26, 95 % CI, −0.17 - -0.008, *P* < 0.05), total body water (standardized β = −0.27, 95 % CI, −0.23 - -0.04, *P* < 0.01), appendicular muscle mass (standardized β = −0.29, 95 % CI, −0.36 - -0.04, *P* < 0.05) and the AMI (standardized β = −0.24, 95 % CI, −1.18 - -0.20, *P* < 0.01) were negatively correlated with the VAS of neck and shoulder pain, whereas no significant correlation was observed between the VAS of back pain, low back pain and any body composition parameters.

**Table 5:** Association between visual analogue scale of present pain and body composition parameters

	Model 1^a^			Model 2^b^		
	Standardised β	95 % CI	*P*-value*	Standardaised β	95 % CI	*P*-value*
Neck and shoulder pain						
Age (y)	−0.16	−0.06 - -0.01	**<0.01**	-	-	-
Total body muscle mass (kg)	−0.13	−0.09 -0.007	**<0.05**	−0.26	−0.17- -0.008	**<0.05**
Total body fat mass (kg)	0.03	−0.04 - 0.72	0.58	−0.007	−0.06 - 0.05	0.91
Total body water (kg)	−0.17	−0.15 - -0.03	**<0.01**	−0.27	−0.23 - -0.04	**<0.01**
Total body fat ratio (%)	0.09	−0.009 - 0.07	0.13	0.04	−0.03 - 0.06	0.57
Total body water ratio (%)	−0.15	−0.14 - -0.01	**<0.05**	−0.11	−0.13 0.007	0.07
Appendicular muscle mass (kg)	−0.13	−0.17 -0.008	**<0.001**	−0.29	−0.36 - -0.04	**<0.05**
Appendicular fat mass (kg)	0.05	−0.08 - 0.20	0.39	−0.02	−0.19 - 0.12	0.67
Trunk muscle mass (kg)	−0.13	−0.16 - -0.01	**<0.05**	−0.16	−0.25 - 0.04	0.16
Trunk fat amount (kg)	0.02	−0.07 - 0.10	0.73	0.004	−0.08 - 0.09	0.94
BMI (kg/m^2^)	−0.08	−0.19 - 0.03	0.16	−0.05	−0.16 - 0.05	0.33
AMI (kg/m^2^)	−0.16	−0.81 - -0.14	**<0.01**	−0.24	−1.18 -0.20	**<0.01**
BMR (kcal)	−0.11	−0.003 - 0.00	0.05	−0.19	−0.005 - 0.000	0.05
Back pain						
Age (y)	−0.09	−0.033 - 0.004	0.11	-	-	-
Total body muscle mass (kg)	−0.02	−0.03 - 0.02	0.74	−0.12	−0.09 - 0.03	0.31
Total body fat mass (kg)	−0.01	−0.04 - 0.03	0.83	−0.01	−0.05 - 0.03	0.79
Total body water (kg)	−0.05	−0.06 - 0.02	0.41	−0.15	−0.12 - 0.01	0.12
Total body fat ratio (%)	0.01	−0.02 - 0.03	0.84	0.02	−0.03 - 0.04	0.75
Total body water ratio (%)	−0.05	−0.07 - 0.02	0.33	−0.06	−0.08 - 0.02	0.31
Appendicular muscle mass (kg)	−0.01	−0.06 - 0.05	0.80	−0.13	−0.18 - 0.05	0.25
Appendicular fat mass (kg)	−0.002	−0.106 - 0.103	0.98	−0.02	−0.13 - 0.09	0.75
Trunk muscle mass (kg)	−0.02	−0.06 - 0.04	0.69	−0.08	−0.15 - 0.07	0.47
Trunk fat amount (kg)	−0.02	−0.07 - 0.05	0.74	−0.01	−0.07 - 0.06	0.81
BMI (kg/m^2^)	−0.04	−0.11 - 0.05	0.48	−0.03	−0.10 - 0.06	0.61
AMI (kg/m^2^)	−0.033	−0.31 - 0.17	0.58	−0.09	−0.57 - 0.16	0.27
BMR (kcal)	−0.01	−0.001 - 0.001	0.76	−0.1	−0.003 - 0.001	0.29
Low back pain						
Age (y)	0.06	−0.01 - 0.03	0.28	-	-	-
Total body muscle mass (kg)	0.02	−0.02 - 0.04	0.67	0.03	−0.06 - 0.08	0.79
Total body fat mass (kg)	−0.05	−0.07 - 0.02	0.34	−0.05	−0.07 - 0.03	0.41
Total body water (kg)	0.02	−0.04 - 0.06	0.69	0.01	−0.07 - 0.09	0.84
Total body fat ratio (%)	−0.04	−0.04 - 0.02	0.44	−0.05	−0.06 - 0.03	0.52
Total body water ratio (%)	0.05	−0.03 - 0.08	0.37	0.04	−0.04 - 0.08	0.47
Appendicular muscle mass (kg)	0.03	−0.05 - 0.09	0.58	0.09	−0.08 - 0.20	0.43
Appendicular fat mass (kg)	−0.06	−0.19 - 0.06	0.30	−0.04	−0.19 - 0.90	0.47
Trunk muscle mass (kg)	0.01	−0.05 - 0.08	0.77	−0.03	−0.15 - 0.11	0.79
Trunk fat amount (kg)	−0.05	−0.11 - 0.04	0.38	−0.05	−0.12 - 0.04	0.39
BMI (kg/m^2^)	−0.03	−0.12 - 0.07	0.60	−0.04	−0.13 - 0.06	0.49
AMI (kg/m^2^)	0.03	−0.22 - 0.37	0.61	0.04	−0.34 - 0.54	0.65
BMR (kcal)	0.01	−0.001 - 0.002	0.83	0.01	−0.002 - 0.003	0.90

BMI, body mass index; AMI, appendicular muscle index; BMR, basal metabolic rate; CI, confidence interval. ^a^simple linear regression model; ^b^multiple linear regression model adjusted for age and gender; *Significant *P*-values are in bold

## Discussion

In the present study, we found some association between body composition and neck and shoulder pain (*katakori* in Japanese) among Japanese subjects, which has not previously been reported, to the best of our knowledge. Despite the fact that there are some limitations in the present study, these findings regarding neck and shoulder pain in the Japanese population provide a new approach to elucidate its pathology.

Regarding neck and shoulder pain in the Japanese population, some associated factors apart from body composition have been previously demonstrated, specifically gender, psychological stress and some types of musculoskeletal pain, to be associated with neck and shoulder pain in Japanese subjects [1, 4, 6, 7]. Furthermore, the association between neck and shoulder pain and sagittal spinal alignment has also been reported [7]. In addition, Fujii et al. reported a significant association between *katakori* and the lack of worksite support from a colleague or supervisor [2]. On the other hand, regarding the association of body composition parameters with neck pain, Yalcinkaya et al. investigated whole-body physical fitness parameters including body composition in Turkish patients with chronic neck pain and healthy controls, and reported that the body fat percentage was higher in male patients with chronic neck pain [22]. However, all subjects with chronic neck pain in their study were patients who had been referred to a department of rehabilitation, whereas the subjects in the present study were from the general population. In addition, they did not investigate the association between pain intensity and body composition, unlike the method adopted in the present study. In the present study, we demonstrated that a smaller percentage in the body water ratio was associated with the presence of neck and shoulder pain and that total body muscle mass, appendicular muscle mass, and the AMI were negatively correlated with the intensity of neck and shoulder pain according to the multivariate analyses. Interestingly, no parameters of the trunk were correlated with the intensity of neck and shoulder pain, in contrast to the correlation between the appendix parameters and neck and shoulder pain. Conversely, we demonstrated that no parameters regarding the body composition were associated with chronic neck and shoulder pain. According to these results regarding neck and shoulder pain, we speculated that the decreased percentage of total body water, that is the dehydration trend, is directly or indirectly associated with the pathology of neck and shoulder pain, although it does not influence the chronicity of neck and shoulder pain. Furthermore, we also speculated that muscle loss, especially appendicular muscle loss with the fat condition remaining relatively stable, may lead to an increased load on the structure around the neck and shoulder during ADL and result in the onset of neck and shoulder pain in Japanese subjects, in contrast to previous reports in which the fat condition is associated with low back pain or neck pain [7, 20, 22]. However, younger age was associated with the presence of neck and shoulder pain, and also age was negatively correlated with the intensity of neck and shoulder pain, despite the fact that the muscle loss was correlated with the intensity of neck and shoulder pain. This fact suggests the possibility that the muscle loss in neck and shoulder pain was not associated with age-related muscle loss (i.e., sarcopenia [35, 36]). More detailed research is necessary to understand fully the relationship between neck and shoulder pain and muscle loss.

On the other hand, regarding the association between low back pain and body composition, Urquhart et al. reported that greater fat mass, but not lean tissue mass, was associated with higher levels of low back pain intensity and disability [19]. Furthermore, Hodselmans et al. reported that the body fat percentage of chronic low back pain patients was significantly higher compared with the normative data [20]. Hicks et al. investigated the associations between the trunk muscle composition and physical function, and they concluded that the subjects with higher low back pain severity had lower muscle attenuation, however, there was no difference in the average trunk muscle area according to the low back pain status [21]. In the present study of Japanese subjects, neither a significant association nor a significant correlation was observed between back pain, low back pain and the body composition parameters. We speculated that the ethnic difference may explain the differences in the results of the present study and the previous studies concerning the relationship between low back pain and body composition. Furthermore, it was possible that low back pain in Japanese subjects is mainly derived not from soft tissues, including fat and muscle, but from bone and/or joints by an anatomical point of view.

The present study has some limitations. First, a clear definition of each type of pain was not adopted; for instance, we did not use an illustration that defined the area for each type of pain in the present study. Therefore, the criteria of reply for the question regarding the presence or absence of neck and shoulder pain may be different depending on the individual. Second, the present study was conducted using data from the annual medical examinations. Therefore, the subjects who worry about their own health condition may comprise the majority of subjects participating in the present study; in other words, the subjects in the present study may not reflect the characteristics of the total population. Third, the present study is a cross-sectional study and is also an exploratory one. Therefore, it is considered that further studies including a prospective study are needed to confirm the findings of several associations between neck and shoulder pain and the body composition obtained in the present study.

## Conclusions

We herein investigated the association between neck and shoulder pain, back pain, low back pain and body composition in the Japanese general population and demonstrated the possibility that some body composition parameters regarding body water and body muscle were associated with the pathology of neck and shoulder pain.

## Abbreviations

QOL: Quality of life
ADL: Activities of daily living
VAS: Visual analogue scale
BIA: Bioelectrical impedance analysis
AMI: Appendicular muscle mass index
BMI: Body mass index
BMR: Basal metabolic rate
OR: Odds ratio
CI: Confidence interval
